# ERRFI1 induces apoptosis of hepatocellular carcinoma cells in response to tryptophan deficiency

**DOI:** 10.1038/s41420-021-00666-y

**Published:** 2021-10-04

**Authors:** Mingqing Cui, Dan Liu, Wujun Xiong, Yugang Wang, Jun Mi

**Affiliations:** 1 Basic Medical Institute; Hongqiao International Institute of Medicine, Tongren Hospital; Key Laboratory of Cell Differentiation and Apoptosis of Chinese Ministry of Education, Shanghai Jiao Tong University School of Medicine, Shanghai, China; 2 Department of Gastroenterlogy, Shanghai Pudong Hospital, Fudan University Pudong Medical Center, Shanghai, China; 3 Department of gastroenterology, Tongren Hospital, Shanghai Jiao Tong University School of Medicine, Shanghai, China

**Keywords:** Cancer metabolism, Tumour biomarkers

## Abstract

Tryptophan metabolism is an essential regulator of tumor immune evasion. However, the effect of tryptophan metabolism on cancer cells remains largely unknown. Here, we find that tumor cells have distinct responses to tryptophan deficiency in terms of cell growth, no matter hepatocellular carcinoma (HCC) cells, lung cancer cells, or breast cancer cells. Further study shows that ERRFI1 is upregulated in sensitive HCC cells, but not in resistant HCC cells, in response to tryptophan deficiency, and ERRFI1 expression level positively correlates with HCC patient overall survival. ERRFI1 knockdown recovers tryptophan deficiency-suppressed cell growth of sensitive HCC cells. In contrast, ERRFI1 overexpression sensitizes resistant HCC cells to tryptophan deficiency. Moreover, ERRFI1 induces apoptosis by binding PDCD2 in HCC cells, PDCD2 knockdown decreases the ERRFI1-induced apoptosis in HCC cells. Thus, we conclude that ERRFI1-induced apoptosis increases the sensitivity of HCC cells to tryptophan deficiency and ERRFI1 interacts with PDCD2 to induce apoptosis in HCC cells.

## Introduction

Hepatocellular carcinoma (HCC) is the fifth most common malignant tumor globally and the second leading cause of cancer-related death [1]. Although surgery is still the primary method for HCC, target therapy on various signaling pathways has been applied in clinical practice [2]. However, drug resistance and tumor heterogeneity limit the efficacy of these drugs, resulting in transient response or even no response to these therapies [3, 4]. Therefore, fully understanding the mechanism underlying HCC initiation and progression is a prerequisite for improving the therapeutic effect of HCC.

Tryptophan, an essential amino acid, is primarily decomposed through the kynurenine pathway in the liver [5]. Tryptophan participates in protein synthesis and plays a vital role in immune regulation, including inflammation, infection, and pregnancy. Recent studies have shown that tryptophan metabolism increases significantly in tumor cells, promoting tumorigenesis and immune evasion [6]. Both tryptophan deficiency and kynurenine accumulation induce apoptosis of the effector T cells and promote differentiation of immunosuppressive Treg cells [7, 8]. Tryptophan deficiency also leads to the dysfunction of T cells by activating the general control non-derepressible 2 (GCN2) kinase pathway [9]. However, the effect of tryptophan metabolism on cancer cells remains largely unknown.

ERRFI1 (ERBB receptor feedback inhibitor 1) is an early response gene encoding a non-kinase scaffold adaptor protein induced by various stimuli such as hormones and stresses [10]. ERRFI1 is considered a negative regulator of EGFR because it can directly bind to EGFR, inhibit the catalytic activity of EGFR, and mediate EGFR lysosomal degradation [11–13]. Considering the involvement of ERRFI1 in the signaling of the EGFR family, its potential role in cancer draws great attention. Studies indicate that ERRFI1 is downregulated in lung cancer, breast cancer, thyroid cancer, and many other cancer types [14–16]. Loss of ERRFI1 not only promotes lung cancer cell proliferation and migration through the ERK pathway in vitro, but also causes spontaneous lung tumorigenesis in mice [14, 17]. ERRFI1 is reported to induce apoptosis and inhibit proliferation and invasion in endometrial cancer, breast cancer, and thyroid cancer, suggesting ERRFI1 functions as a tumor suppressor in certain cancers [15, 16, 18, 19]. Nevertheless, the role of ERRFI1 in HCC is not fully clarified.

PDCD2 (Programmed cell death domain 2) encoding a nuclear protein expresses in a variety of tissues. PDCD2 is originally discovered in a screen for genes associated with apoptosis in rat cells [20]. It is generally accepted that PDCD2 is critical to stemming cell differentiation and embryonic development [21]. In addition, the aberrant expression of PDCD2 is relevant to the development of cancer. PDCD2 expresses at a low level in osteosarcoma and gastric cancer, and the knockdown of PDCD2 promotes cancer cell proliferation [22, 23]. However, PDCD2 is highly expressed in human acute leukemia cells and PDCD2 overexpression facilitates cancer cell growth [24]. Therefore, the biological function of PDCD2 in tumor is controversial and needs to be studied intensively.

In the present study, the effect of tryptophan deficiency on HCC cells is studied, and the potential mechanism underlying ERRFI1 inducing apoptosis is further investigated.

## Results

### Tryptophan deficiency suppresses the growth of some cancer cells

In order to investigate the role of tryptophan in cancer cells, several types of cancer cells were growing in the media absence of tryptophan. The cytometry analysis showed that tryptophan deficiency inhibited cell growth and reduced cell numbers of specific cancer cells, regardless of cancer types.

**Fig. 1:**
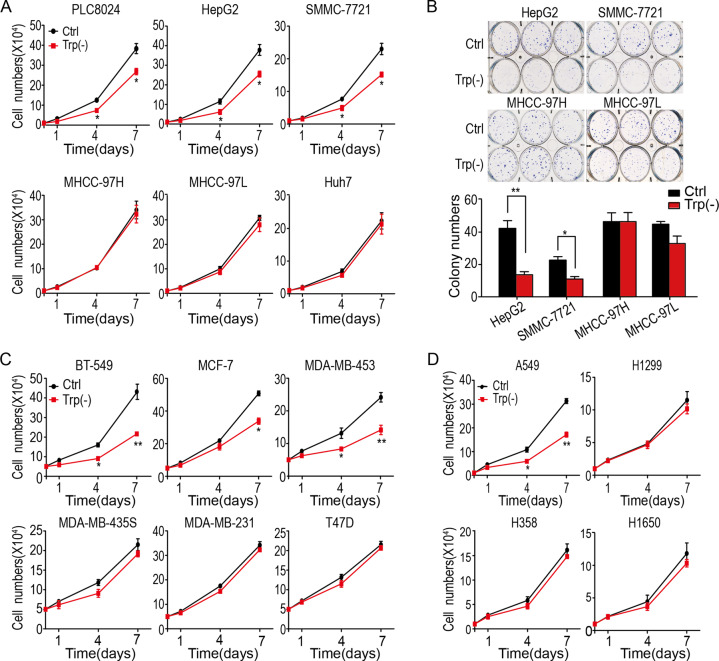
Tryptophan deficiency suppresses the growth of some cancer cells. **A** The effects of tryptophan deficiency on HCC cells growth were analyzed by cytometry assay. **P < 0.01; *P < 0.05. **B** The effects of tryptophan deficiency on HCC cells growth were analyzed by colony formation assay. **P < 0.01; *P < 0.05. **C** The effects of tryptophan deficiency on breast cancer cells growth were analyzed by cytometry assay. **P < 0.01; *P < 0.05. **D** The effects of tryptophan deficiency on lung cancer cells growth analyzed by cytometry assay. **P < 0.01; *P < 0.05. Trp tryptophan.

As shown in Fig. 1A, hepatocellular carcinoma (HCC) PLC8024, HepG2, and SMMC-7721 cells were sensitive to tryptophan deficiency. In contrast, the other HCC cells, such as MHCC-97H, MHCC-97L, and Huh7 cells, were resistant to tryptophan deficiency, which had little effect on the growth of these HCC cells. In agreement with the finding, colony formation assay demonstrated that tryptophan deficiency significantly decreased colony numbers of HepG2 and SMMC-7721 cells, but not MHCC-97H and MHCC-97L cells (Fig. 1B), suggesting the sensitivity of HCC cells to tryptophan deficiency is varied, and the PLC8024, HepG2, and SMMC-7721 cells were sensitive to tryptophan deficiency. In contrast, MHCC-97H, MHCC-97L, and Huh7 cells were tryptophan deficiency-resistant cells.

Furthermore, the sensitivity of the other two types of cancer cells to tryptophan was examined, including breast cancer cells and lung cancer cells. As shown in Fig. 1C, D, tryptophan deficiency only inhibited the cell growth and reduced cell numbers of part of breast cancer cells and lung cancer cells, suggesting the differences in the sensitivity of cancer cells to tryptophan deficiency are common in various cancer cells.

### The expression of ERRFI1 is associated with the prognosis of HCC

**Fig. 2:**
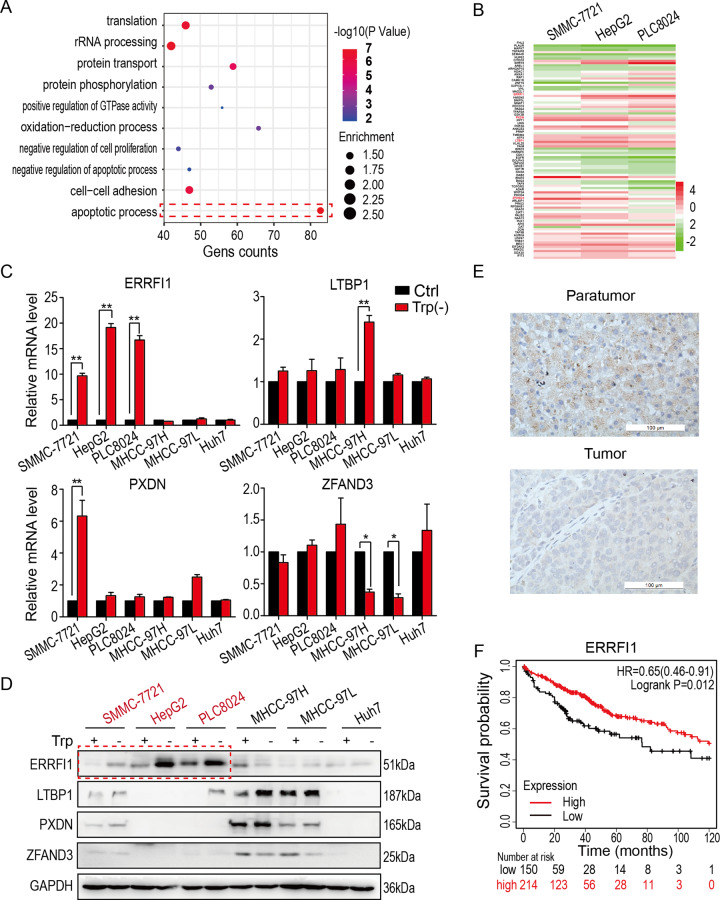
The expression of ERRFI1 is positively correlated with prognosis in HCC. **A** Gene Ontology (GO) analysis on the commonly upregulated genes in three sensitive HCC cells (SMMC-7721, HepG2, and PLC8024) in response to tryptophan deficiency. **B** The mRNA expression heatmap of genes involved in apoptosis signaling in response to tryptophan deficiency. **C**, **D**. Expression detection of candidate genes including ERRFI1, LTBP1, PXDN, and ZFAND3 by qPCR and western blot in HCC cells with or without tryptophan supplementation. **P < 0.01; *P < 0.05. **E** Immunohistochemistry analysis of ERRFI1 on clinical HCC samples. The paratumor tissue was considered as a control. Scale bar = 100 µm. **F** Kaplan–Meier analysis on ERRFI1 expression with overall survival of HCC patients.

To explore the mechanism by which HCC cells are sensitive to tryptophan deficiency, we first carried RNA-sequencing analysis in six HCC cell lines, including SMMC-7721, HepG2, PLC8024, MHCC-97H, MHCC-97L, and Huh7. Then gene ontology analysis was performed on the differentially expressed genes between the tryptophan deficiency-sensitive and -resistant HCC cells. As shown in Fig. 2A, apoptosis signaling was the most significant pathway activated in the tryptophan deficiency-sensitive HCC cells. Furthermore, the differentially expressed genes were displayed by heatmap in SMCC-7721, HepG2, and PLC8024, three tryptophan-sensitive HCC cell lines. ERRFI1, LTBP1, PXDN, and ZFAND3, the four most upregulated genes in three sensitive cell lines, were chosen for candidate genes (Fig. 2B).

Moreover, the expression of four candidate genes was individually verified by quantitative PCR in six HCC cell lines, including the tryptophan deficiency-sensitive and -resistant HCC cells. The expression was individually normalized to their basal expression in cells supplemented with tryptophan, and only ERRFI1 expression increased significantly in PLC8024, HepG2, and SMMC-7721, three sensitive HCC cells, but not in MHCC-97H, MHCC-97L, and Huh7, three resistant cells (Fig. 2C), which was in line with the RNA-sequencing data. However, the expression of LTBP1, PXDN, and ZFAND3 in HCC cells was not consistent with the RNA-sequencing data. These results were further confirmed by western blot analysis, which showed that only ERRFI1 protein levels increased in PLC8024, HepG2, and SMMC-7721 cells in response to tryptophan deficiency (Fig. 2D), suggesting that ERRFI1 expression upregulates in sensitive HCC cells in response to tryptophan deficiency.

In addition, the immunohistochemistry staining showed that ERRFI1 was significantly downregulated in HCC samples compared to para-tumor tissues (Fig. 2E). Also, the Kaplan–Meier survival analysis showed that HCC patients with higher ERRFI1 expression had more prolonged overall survival than those with lower ERRFI1 expression (*P* < 0.05, Fig. 2F), suggesting that the expression level of ERRFI1 is positively correlated with prognosis in HCC.

### ERRFI1 induces apoptosis of HCC cells in response to tryptophan deficiency

**Fig. 3:**
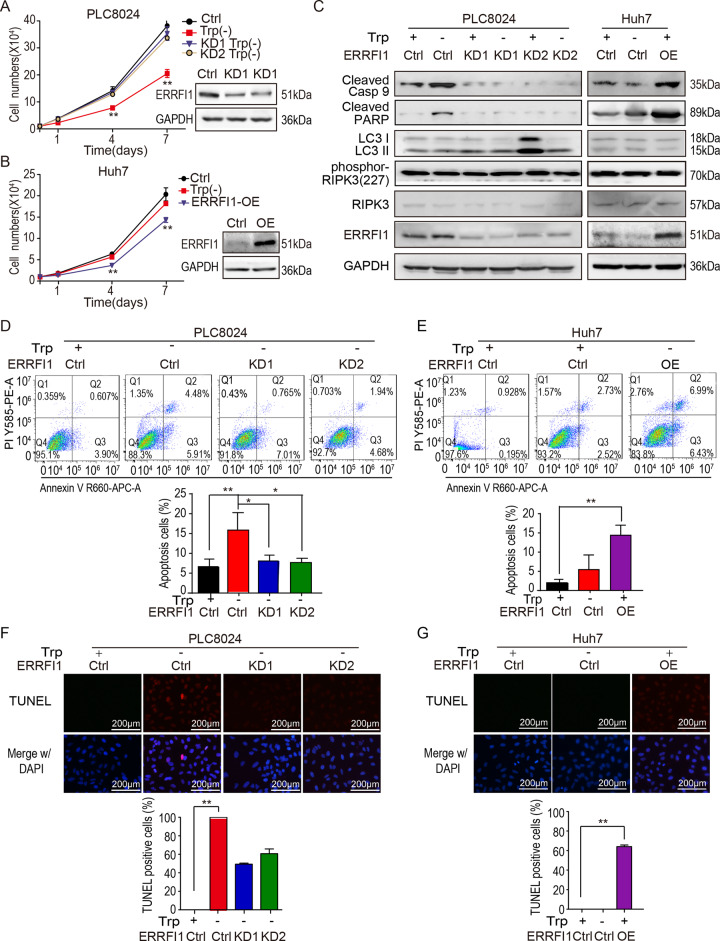
ERRFI1 mediates tryptophan deficiency-suppressed HCC cells growth. **A** The effect of ERRFI1 knockdown on cell growth in sensitive PLC8024 cells by cytometry assay and western blot (**P < 0.01; *P < 0.05). **B** The effect of ERRFI1 overexpression on cell growth in resistant Huh7 cells by cytometry assay and western blot (**P < 0.01; *P < 0.05). **C** Detection of apoptosis markers in the sensitive PLC8024 cells depleted of ERRFI1 or in the resistant Huh7 cells overexpressing ERRFI1 with or without tryptophan supplementation by western blot. **D**, **E** The apoptosis assessment by flow cytometry assay in the ERRFI1 knockdown PLC8024 cells and the ERRFI1 overexpressed Huh7 cells with or without tryptophan supplementation. **F**, **G** The apoptosis assessment by TUNEL assay in the ERRFI1 knockdown PLC8024 cells and the ERRFI1 overexpressed Huh7 cells with or without tryptophan supplementation. Scale bar = 200 µm.

According to the above finding that the expression of ERRFI1 associates with the sensitivity of HCC cells to tryptophan, we speculate whether ERRFI1 suppresses HCC cells growth in response to tryptophan deficiency. The cytometry analysis showed that tryptophan deficiency inhibited PLC8024 cells growth, and depletion of ERRFI1 rescued the tryptophan deficiency-suppressed cell growth (Fig. 3A). Meanwhile, ERRFI1 overexpression decreased cell growth of Huh7 cells, which were resistant to tryptophan deficiency, suggesting ERRFI1 promotes HCC cell death (Fig. 3B).

It is widely accepted that three mechanisms lead to cell death, including apoptosis, autophagy, and necroptosis [25, 26]. In order to identify the pathway by which ERRFI1 promotes cell death, well-accepted markers for three mechanisms were examined in HCC cells by western blot analysis. As shown in Fig. 3C, tryptophan deficiency increased the cleaved Casp-9 and PARP levels, but not the LC3 II or phospho-RIPK3 in the sensitive PLC8024 cells. ERRFI1 knockdown abolished this enhancement. In contrast, tryptophan deficiency did not change the level of these markers in the resistant Huh7 cells, including cleaved Casp-9 and PARP. And ERRFI1 overexpression promoted the Casp-9 and PARP cleavage, indicating that tryptophan deficiency inhibits HCC cells growth by inducing apoptosis.

In line with the finding, both flow cytometry analysis and TUNEL assay confirmed that tryptophan deficiency increased the subpopulation of apoptotic PLC8024 cells, and ERRFI1 knockdown decreased the cell number of apoptotic PLC8024 cells in response to tryptophan deficiency (Fig. 3D,F). In contrast, ERRFI1 overexpression increased the subpopulation of apoptotic Huh7 cells from approximately 5% to 13% (Fig. 3E, G). The control Huh7 cells were resistant to tryptophan deficiency. Collectively, these findings demonstrated that ERRFI1 plays an essential role in tryptophan deficiency-induced HCC cell apoptosis.

### ERRFI1 interacts with PDCD2 to induce apoptosis

**Fig. 4:**
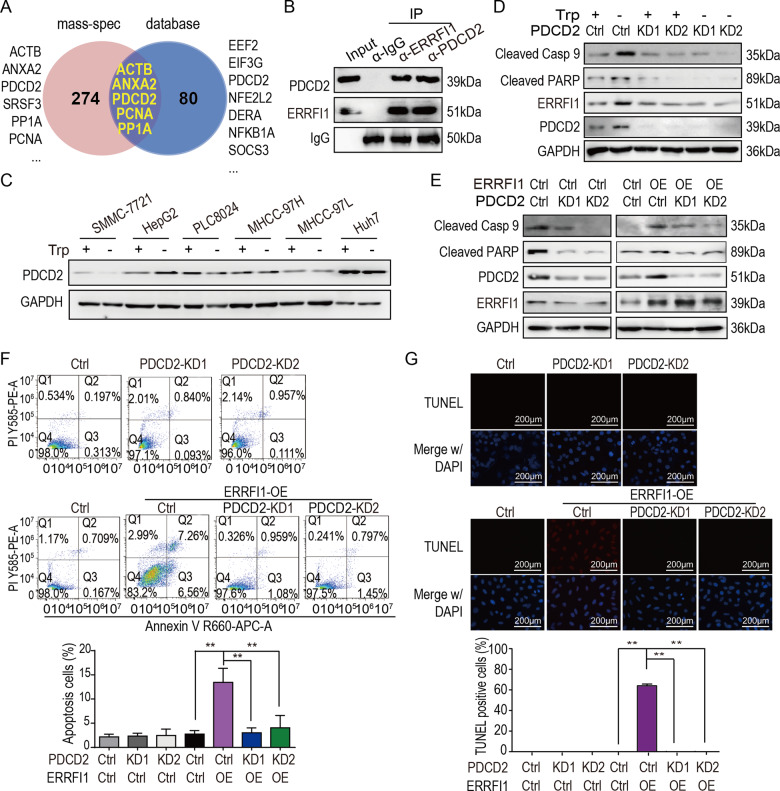
ERRFI1 interacts with PDCD2 to induce apoptosis on HCC cells. **A** The mass-spectrometry identified protein overlapped with the ERRFI1 binding proteins from the online database. **B** PDCD2 detection following co-immunoprecipitation of ERRFI1. **C** PDCD2 protein detection in HCC cells with or without tryptophan supplementation. **D** Detection of apoptosis markers in the sensitive PLC8024 cells depleted of PDCD2 with or without tryptophan supplementation. **E** Detection of apoptosis markers in the resistant Huh7 cells depleted of PDCD2 or overexpressing ERFFI1. **F**, **G** The apoptosis assessment by flow cytometry and TUNEL assay in Huh7 cells depleted of PDCD2 or overexpressing ERFFI1.Scale bar = 200 µm.

Protein mass-spectrometry analysis was performed to determine the mechanism by which ERRFI1 induces apoptosis in HCC cells, following the co-immunoprecipitation against ERRFI1. The mass-spectrometry study identified 274 candidate proteins binding ERRFI1, and 5 proteins were overlapping with identified ERRFI1 binding protein from the online database (thebiogrid.org) (Fig. 4A). Among them, PDCD2 is an apoptosis regulation protein [21, 27, 28].

The western blot following co-immunoprecipitation was performed to validate the association of ERRFI1 with PDCD2. As shown in Fig. 4B, PDCD2 co-immunoprecipitated with ERRFI1, suggesting PDCD2 forms a complex with ERRFI1. Moreover, the expression of PDCD2 was analyzed in HCC cells in response to tryptophan deficiency. Indeed, the expression of PDCD2 was not changed in HCC cells with or without tryptophan, no matter in sensitive HCC cells or in resistant HCC cells (Fig. 4C). Then, PDCD2 was knocked down to determine whether PDCD2 engaged in ERRFI1-induced apoptosis. As shown in Fig. 4D, PDCD2 knockdown reduced the cleaved Caspase 9 and PARP levels, two apoptosis markers, in the sensitive PLC8024 cells in response to tryptophan deficiency. Moreover, PDCD2 knockdown also decreased the cleaved Caspase 9 and PARP levels in resistant Huh7 cells overexpressing ERRFI1 (Fig. 4E).

To further determine whether PDCD2 mediates ERRFI1-induced apoptosis, the flow cytometry analysis and Tunnel assay were performed. As shown in Fig. 4F, PDCD2 knockdown decreased the subpopulation of ERRFI1-induced apoptotic Huh7 cells while PDCD2 knockdown has no significant effect on the apoptosis of control Huh7 cells. Consistent with this finding, the Tunnel assay showed that PDCD2 knockdown decreased the ERRFI1-induced apoptotic foci in Huh7 cells (Fig. 4G). Collectively, these findings demonstrated that ERRFI1 interacts with PDCD2 to induce apoptosis in HCC cells.

## Discussion

Tryptophan plays a crucial role in maintaining the tumor immunosuppressive microenvironment. Previous studies have shown that tryptophan deficiency and kynurenine accumulation have substantial immunoregulatory effects on the tumor microenvironment. This microenvironment leads to tumor immune escape [6, 29]. For example, the expression of IDO, the key enzyme of tryptophan metabolism, is elevated in several cancers including ovarian cancer, breast cancer, and glioma [30]. The upregulation of IDO results in increased T cell infiltration and inflammation by reducing the levels of tryptophan and activating GCN2 [31]. In addition, the upregulation of IDO increases the accumulation of kynurenine, the intermediate metabolite of tryptophan metabolism. Aryl hydrocarbon receptor (AhR) activation by kynurenine results in the generation of immune-tolerant dendritic cells and regulatory T cells, which foster a tumor immunological microenvironment [32]. While the immunological regulation of tryptophan metabolism on tumor has been well studied, the effect of tryptophan on cancer cells themselves is poorly understood. Therefore, we focus on the effect of tryptophan deficiency on HCC cells. In this study, the HCC cells showed a distinct response to tryptophan deficiency as reflected by cell growth. We extended the results obtained in HCC cells to a panel of other cancer cells and confirmed that cancer cells showed a distinct response to tryptophan deficiency.

ERRFI1 was a potential gene that could be accounted for the distinct response through an RNA-sequencing screen. ERRFI1 is a cytoplasmic protein whose expression is upregulated with cell growth [33]. We confirmed that ERRFI1 expression increased significantly in tryptophan deficiency-sensitive HCC cells. Moreover, the apoptosis pathway was greatly activated in tryptophan deficiency-sensitive HCC cells according to bioinformatics analysis, suggesting tryptophan deficiency may induce apoptosis of HCC cells by upregulating the expression of ERRFI1. In consistent with other reports [34], we found that the expression of ERRFI1 was downregulated in HCC and low expression of ERRFI1 predicted a poor prognosis, indicating that ERRFI1 is willing to be a biological marker of HCC prognosis.

Functionally, the specific role of ERRFI1 in HCC cells was corroborated by flow cytometry analysis and TUNEL assays. Further investigation verified that overexpression of ERRFI1 promoted HCC cell death while knockdown of ERRFI1 rescued the tryptophan deficiency-suppressed cell growth. Western blot analysis further confirmed the results at the protein level, supporting our hypothesis that ERRFI1 induces apoptosis of HCC cells in response to tryptophan deficiency.

ERRFI1 is induced by various stimuli such as hormones and stresses [10]. It is reported that ERRFI1 decreases tumor formation by inhibiting cell proliferation and increasing apoptosis [12, 19, 34]. ERRFI1 has been shown to promote apoptosis in lung cancer cells via the ERK pathway [19]. Here, we report for the first time that ERRFI1 and PDCD2 formed a complex in HCC cells. PDCD2 is considered to be related to cell death and PDCD2 could suppress sorafenib-resistant HepG2 cells from undergoing EMT and promote apoptosis [21]. Our findings provide the first proof of concept that ERRFI1 interacted with PDCD2 to induce apoptosis, suggesting ERRFI1 induces apoptosis by various mechanisms. The way how ERRFI1 binds with PDCD2 still needs to be further investigated.

In brief, our results reveal that ERRFI1 induces apoptosis in HCC cells in response to tryptophan deficiency and low expression of ERRFI1 predicts a poor prognosis, indicating that ERRFI1 is a potential prognostic marker for HCC.

## Methods & materials

### Cells and reagents

*Human HCC cells PLC8024, HepG2, SMMC-7721, MHCC-97H, MHCC-97L, and Huh7, human breast cancer cells BT-549, MCF-7, MDA-MB-453, MDA-MB-435S, MDA-MB-231, and T47D, human lung cancer cells A549, H1299, H358, and H1650 were cultured in DMEM (L110KJ, BasalMedia, Shanghai, China) with 10% FBS (10100147, Gibco, NY, USA). Tryptophan-deprived DMEM was purchased from BasalMedia (X004W1, Shanghai, China). The source for antibodies used for western blot was as follows: ERRFI1 (11630-1-AP, Proteintech, IL, USA), LTBP1 (26855-1-AP, Proteintech, IL, USA), PXDN (A17929, Abclonal, Wuhan, China), ZFAND3 (A7478, Abclonal, Wuhan, China), LC3 (14600-1-AP, Proteintech, IL, USA), Cleaved Caspase-9 (9748* *S, CST, MA, USA), PARP (9542* *S, CST, MA, USA), Cleaved PARP (5625* *S, CST, MA, USA), PDCD2 (ab133342, Abcam, Cambridge, UK), Anti-RIPK3 (phospho S227) (ab209384, Abcam, Cambridge, UK), RIPK3 (17563-1-AP, Proteintech, IL, USA), and GAPDH (EM1901-57, Huabio, Hangzhou, China)*.

### Selection of specific gene signatures and functional enrichment analysis

Cells that cultured with tryptophan-deprived or complete DMEM were collected for RNA-Seq. To examine whether genes were enriched in any biological processes potentially relevant to HCC, the functional prediction was performed using Gene Ontology (GO) annotation with cluster profile (v3.8.1), and GO BP terms with an FDR-corrected *P* ≤ 0.05 were considered statistically significant. The correlation between the interest gene expression and the survival time of HCC patients was analyzed in the Kaplan–Meier analysis database (http://kmplot.com/analysis).

### Quantitative real-time PCR

Total RNA was extracted using RNAiso Plus (9108, Takara, Shigaken, Japan) and was reverse transcribed into cDNA by PrimeScript™ RT reagent kit (RR037, Takara, Shigaken, Japan). Quantitative real-time PCR reactions were performed in triplicate using SYBR^®^ Green Realtime PCR Master Mix (QPK-201, TOYOBO, Osaka, Japan). The β-actin gene was used as a control for normalization. The primers sequences were as follows:

ERRFI1, forward 5′-CTGGAGCAGTCGCAGTGAG-3′ and reverse 5′-GCCATTCATCGGAGCAGATTTG-3′; LTBP1, forward 5′- CTGACGGCCACGAACTTCC-3′ and reverse 5′-GCACTGACATTTGTCCCTTGA-3′;

PXDN, forward 5′-AATCAGAGAGATCCAACCTGGG-3’ and reverse 5’-AATGCTCCACTAGGTATCCTCTT-3′; ZFAND3, forward 5′- CCAGACGATGATTCCGCTCC-3′ and reverse 5′- GCGTGGTTATCGAGGTATTGTT-3′; β-actin forward 5′-GCGGGAAATCGTGCGTGACATT-3′ and reverse 5′- GATGGAGTTGAAGGTAGTTTCG-3′.

### Western blot

Cells were harvested and lysed on ice for 20 min in RIPA lysis buffer supplemented with a cocktail of protease inhibitors and PMSF. Protein concentration was determined by a BCA protein quantity kit (BCA02, Dingguo Biotechnology, Beijing, China). Protein lysates were subjected to SDS-PAGE on 12% polyacrylamide gels, blotted onto 0.45 μm PVDF transfer membranes, and blocked with 5% non-fat dry milk in TBST for 1 h. Membranes were incubated with the specific primary antibody at 4 °C overnight and then incubated with a secondary antibody. Membranes were scanned using ImageQuant LAS 4000 (GE, UK).

### Lentiviral shRNA knockdown and gene overexpression

Short-hairpin sequences targeting human PDCD2 (target sequence 5′-CAGATCATCTGGACCATAT-3′) were purchased from Shanghai Jiao Tong University School of Medicine. ERRFI1 over-expression primers were synthesized in BioSune (Shanghai, China). ERRFI1 forward primer: 5′-CTAGCTAGCATGTCAATAGCAGGAGTTGCTGCTCAG -3′, and ERRFI1 reverse primer: 5′-CGCGGATCCCTAAGGAGAAACCACATAGGATAAATGTTTACGC-3′. The plasmids were co-transfected into 293 T cells with psPAX2 and pMD2G to generate the lentiviruses. Viruses were collected from the supernatant of 293 T cells and used to infect cells.

### Flow cytometry

Huh7 cells, which were seeded in 6-well plates, were washed with PBS and stained with Annexin V-APC/PI apoptosis kit (70-AP107-100, Multisciences, Zhejiang, China). Approximately 10^4^ cells were analyzed by a CytoFlex LX (Beckman, CA, USA). Cells were gated based on the forward and scatter characteristics.

### TUNEL assay

TUNEL assay was performed using a kit (C1090, Beyotime, Jiangsu, China) according to the manufacturer’s instructions. Cells were fixed in 4% paraformaldehyde. After permeabilization with Triton X-100 and incubation with methanol, the cells were labeled with dUTP nick. Then the cells were added with DAPI and observed by fluorescence microscopy (Leica DM2500, Germany).

### Immunohistochemistry

Archival paraffin-embedded tissues samples from patients with HCC were collected. Representative formalin-fixed, paraffin-embedded tumor tissue blocks were selected, and 5 µm sections for each lesion were prepared for immunohistochemical analysis. The sections were deparaffinized with xylene, hydrated in serial solutions of alcohol, and heated in a pressure cooker containing sodium citrate for antigen retrieval. Endogenous peroxidase activity blocking was performed using 3% hydrogen peroxide solution containing sodium azide for 10 min. The sections were blocked with 10% goat serum for 1 h and incubated with primary antibody overnight at 4 °C. After incubation with horseradish peroxidase-conjugated polyclonal antibody, tissues were visualized with a DAB substrate (CSB-K09758AB, Dingguo Biotechnology, Beijing, China). The sections were then counterstained with Meyer’s hematoxylin, dehydrated, coverslipped, and observed by microscopy.

### Immunofluorescence staining

Cells were seeded on coverslips placed in the bottom of 6-well multi-plates. After the treatments, cells were fixed with 4% paraformaldehyde for 30 min and washed in PBS. After permeabilization with 0.2% Triton X-100 (in PBS) for 10 min, the cells were blocked with 10% BSA for 30 min. Then the cells were incubated with primary antibodies overnight at 4 °C. After incubation with fluorescence conjugated secondary antibodies, the cells were stained with DAPI and observed by fluorescence microscopy.

### Mass spectrometry

Cells were harvested in 0.3% NP40 buffer with a protease inhibitor cocktail prior to centrifugation at 15,000 g for 45 min. Undiluted anti-ERRFI1 serum was exposed to supernatants (1:100 w/w) for 1 h under constant agitation. Protein A/G PLUS-Agarose (sc-2003, Santa Cruz Biotechnology, CA, USA) was added to the sample (1:1 v/v) and incubated under agitation for 30 min. Following several rinses in buffer, protein lysates were analyzed by TripleTOF^TM^ 5600 + (AB SCIEX, USA).

### Co-immunoprecipitation

Samples were homogenized in a single detergent cleavage solution supplemented with a cocktail of protease inhibitors and PMSF. The extracts (1000 µg protein per sample) were pre‐cleared with protein G beads and then mixed with non-specific IgG or anti-ERRFI1 antibody overnight at 4 °C, followed by the addition of 40 µL of protein G beads for 2 h at 4 °C. Immune complexes were washed 2 times and resuspended in 1× SDS loading buffer. The samples were further analyzed by western blot.

### Statistical analysis

All data are presented as means ± SD and are representative of at least three independent experiments. The differences between groups were assessed by Student’s t-test. All reported differences are *P* < 0.05 unless otherwise stated.
